# Novel passive detection approach reveals low breeding season survival and apparent lactation cost in a critically endangered cave bat

**DOI:** 10.1038/s41598-022-11404-4

**Published:** 2022-05-05

**Authors:** Emmi van Harten, Ruth Lawrence, Lindy F. Lumsden, Terry Reardon, Thomas A. A. Prowse

**Affiliations:** 1 Department of Ecology, Environment and Evolution, Research Centre for Future Landscapes, La Trobe University, Bundoora, VIC 3086 Australia; 2 Department of Geography, University of Melbourne, Parkville, VIC 3010 Australia; 3 Department of Environment, Land, Water and Planning, Arthur Rylah Institute for Environmental Research, Heidelberg, VIC 3084 Australia; 4 South Australian Museum, Adelaide, SA 5001 Australia; 5 School of Biological Sciences, The University of Adelaide, Adelaide, SA 5005 Australia

**Keywords:** Ecology, Conservation biology, Population dynamics

## Abstract

Capture-mark-recapture/resight (CMR) methods are used for survival-rate studies, yet are often limited by small sample sizes. Advances in passive integrated transponder (PIT) technology have enabled passive detection or ‘resight’ of marked individuals using large antennas with greater read-ranges than previously possible. We used passively-detected resight data and CMR models to study survival rates of the southern bent-winged bat *Miniopterus orianae bassanii*, a critically endangered, cave-dwelling bat. Over three years, we used PIT-tagging to monitor 2966 individuals at the species’ largest breeding aggregation, using daily detection data (> 1.6 million detections) to estimate seasonal survival probabilities, structured by age, sex and reproductive status, and parameterise population projection matrices. This has hitherto been impossible using traditional CMR methods due to disturbance risk and low recapture rates. Bats exhibited lowest apparent seasonal survival over summer and autumn, particularly for reproductive females in summer (when lactating) and juveniles in autumn (after weaning), and high survival in winter. Lowest survival rates coincided with severe drought in summer–autumn 2016, suggesting that dry conditions affect population viability. Under all likely demographic assumptions, population projection matrices suggested the population is in deterministic decline, requiring urgent action to reduce extinction risk. Passively-collected resight data can now be used in combination with CMR models to provide extensive, robust information for targeted wildlife population management.

## Introduction

Survival analyses are a key tool for understanding wildlife population variation and can provide valuable information for the effective recovery of threatened species^1,2^. Survival-rate studies for terrestrial vertebrates have traditionally relied on labour intensive mark-recapture studies; however, technological advances are providing new opportunities. One technique that can be employed for estimating survival rates in animals uses passive integrated transponder (PIT) tag technology to re-detect tagged individuals at key locations without the need for physical recapture^3^. This method has been demonstrated to significantly increase resight probability and more precisely estimate survival compared to traditional methods^4^. A limitation of PIT technology for undertaking long-term passive mark-resight studies has been the size and read-range capabilities of PIT antennas, with animals needing to pass close (usually *c.* 15 cm) to the readers to be detected, thereby restricting the potential application of this approach for many species. However, PIT-tracking systems have recently become available that can be successfully optimised to detect small bats flying in cave passages up to 2 × 5 m in size^5^.

The use of PIT-tag technology has been recommended for studying insectivorous bats due to challenges such as low recaptures rates^6^ and adverse impacts associated with traditional banding in many species^7^. To date, survival estimates using mark-resight approaches using passively collected data have been published only for a few cool-temperate species, including big brown bats *Eptesicus fuscus*^8–10^ and little brown bats *Myotis lucifugus*^11^ roosting in buildings, lesser short-tailed bats *Mystacina tuberculata* using tree hollows^12,13^, and Daubenton’s bats *Myotis daubentonii* and Natterer’s bats *Myotis nattereri* hibernating in an old well shaft^14^. Undertaking survival analyses using passively collected data for further species in different environments and seasons could provide critical information to inform conservation and recovery of bat populations, which are declining worldwide^15^.

Survival studies of bats have been published for almost a century, but most early studies had methodological issues including reliance on potentially injurious banding and repeated sampling at hibernacula (creating disturbance which may lower bat survival due to the bats burning valuable winter fat stores)^8^. A global synthesis of survival estimates found that bat survival rates were strongly associated with age, sex and the number of young produced per year, as well as additional factors including season, species guild and data collection methods^16^. Survival was highest for adult females in summer and those that produce fewer young per year^16^. First-year survival is commonly lower than annual adult survival^8,16–18^. Several studies have reported higher survival rates in females than males^16,17,19^, while others found no significant difference between the sexes^20–22^. Similarly, a number of studies found no effect of seasonal or climatic effects on bat survival^20,23^, whilst others identified higher survival in summer^16^ or winter^10,14^. CMR studies using traditional mark-recapture methods require intermittent capture periods that often suffer from low recapture rates^6^, so the power to detect such sex- and season-specific differences in survival can be small. Whilst much is yet to be learned about bat demography, bats typically have significantly higher survival rates and slower life histories than expected for their small size^24^.

To investigate the capacity of PIT technology to improve estimates of structured survival rates for small, cave-dwelling bats, our case study focused on the critically endangered southern bent-winged bat *Miniopterus orianae bassanii*, an insectivorous bat with a restricted range in south-eastern Australia. The southern bent-winged bat has undergone serious decline since the 1960s. However, the cause of this historic decline remains uncertain^25^ and the current population trajectory is also unclear. The maximum longevity of the species is at least 20.5 years^26^ and current population mortality does not appear to be due to parasitic or pathogenic factors^27–31^. Survival estimates by age, sex and season are urgently needed to assist managers to identify the most likely population threats, determine the current population trend and understand whether any cohort or time of year is contributing disproportionately to mortality^25^. Historical literature on other Australian bent-winged bats suggest that the cold season may be associated with highest mortality, particularly if individuals are unable to accumulate sufficient fat stores prior to winter^32,33^. We therefore predicted that apparent survival rates would be lowest in winter, particularly for juveniles that probably enter this period with lower fat stores^34^.

Here, we investigate age-, sex- and season-specific survival rates of the southern bent-winged bat over three years, by PIT-tagging and monitoring almost 3,000 individuals at their largest breeding aggregation. We analyse seasonal variation in estimates of apparent survival for the respective age, sex and reproductive classes, consider potential explanations of mortality, calculate population growth rates to assess whether population decline is ongoing, and discuss the benefits and limitations of the PIT methods employed. The results provide essential data for implementing targeted recovery actions for the southern bent-winged bat, and highlights areas that would benefit from further research for analysing rich capture-mark-resight (CMR) datasets in other wildlife species.

## Results

### Capture demographics

A total of 2966 southern bent-winged bats were PIT-tagged, with approximately 1000 bats tagged per year (Table 1). The juvenile sex ratio approached the expected 1:1 in 2016 and 2018 but had statistically significant (*P* < 0.01) male bias in 2017 (Table 1). As expected at a maternity site, adult captures had female bias across all study years. The proportion of adult females classed as lactating ranged between 62.5 and 100% of adult females captured in January capture periods (across study years), suggesting that the majority of adult females bred each year (Supplementary Table S1). Just 28.5% of captured adult females were classed as lactating on a trapping trip on 3 February 2017, suggesting that juveniles were being weaned at this time.

**Table 1:** The number of southern bent-winged bats tagged from each age/sex class (Juv = juvenile, Ad = adult) at Bat Cave, Naracoorte, Australia, 2016–2018.

Year	Capture summary (*n*)					Sex ratio (♂:♀)	
	Juv♂	Juv♀	Ad♂	Ad♀	Total	Juv	Ad
2016	271	282	176	243	972	49:51	42:58
2017	358	292	161	188	999	55:45	46:54
2018	249	269	222	255	995	48:52	46:54

A small number of tagged females recaptured in subsequent years provided some observations of reproductive maturity. Two females tagged as juveniles were recaptured one year after tagging and classed as pre-parous (i.e. non-reproductive). A further two females tagged as juveniles were recaptured two years after tagging and classed as lactating. Whilst the sample size was small, these observations suggest that females may not typically breed until two years of age.

### Body mass

**Figure 1:**
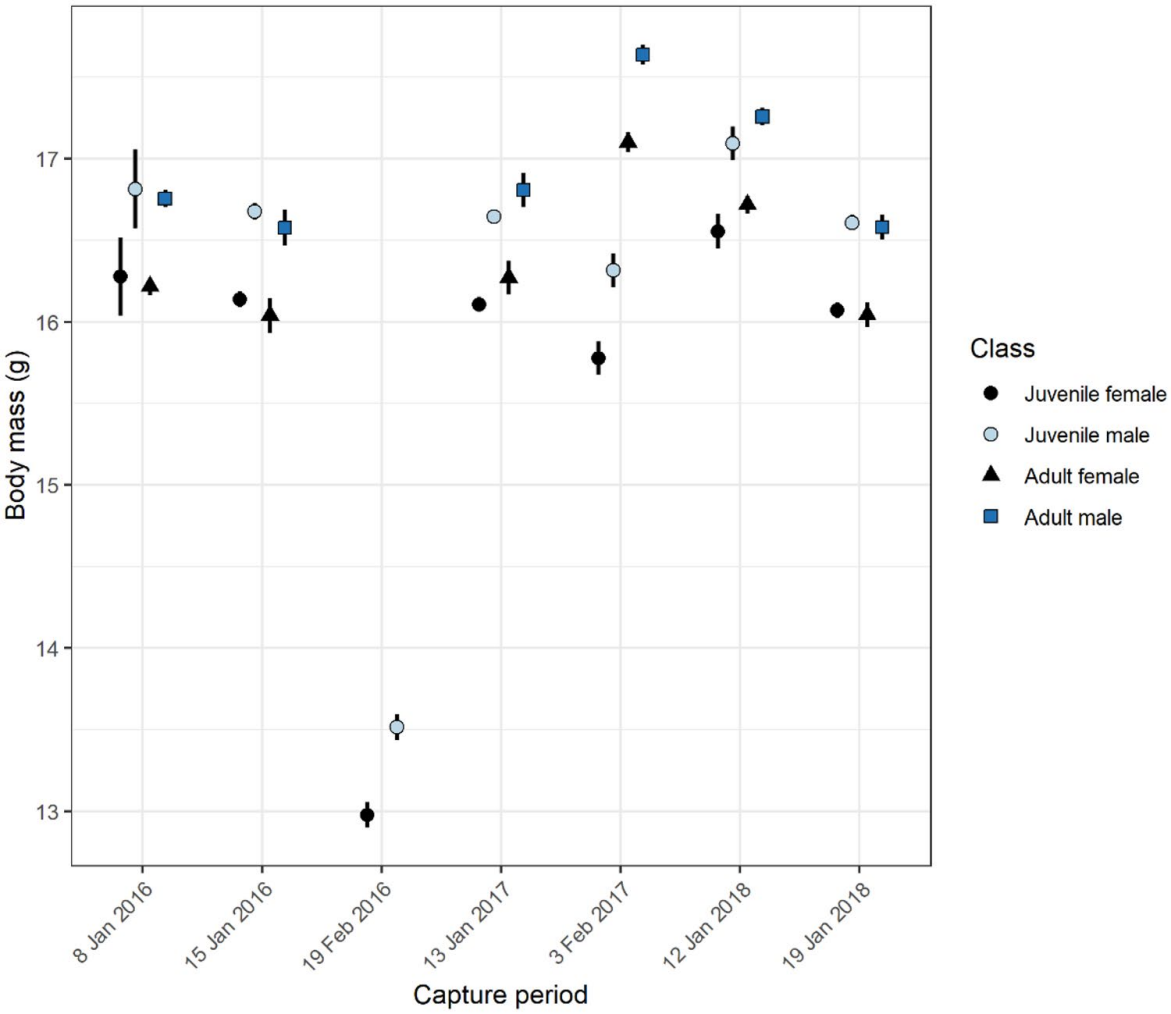
Body mass of southern bent-winged bats for each capture period over the study. Error bars show standard error. Only juveniles were tagged and weighed during the 19 February 2016 capture period, so adult body mass during this period is unknown. Full summary data for body mass is available in Supplementary Table S2.

Sexual dimorphism was evident, with males consistently weighing more than females of the same age cohort (Fig. 1, Supplementary Table S2). In January, juveniles were newly volant, yet already approximated adult proportions and juvenile males exceeded the weight of adult females. Highest body mass in adults was observed on 3 February 2017, which appeared to coincide with weaning and some decrease in juvenile body mass. A capture period in mid-February 2016 after weaning saw a marked decline in body mass for juveniles of both sexes compared to during the previous month and all other capture periods. No adults were tagged (and hence weighed) during this later trip so it is unknown whether adults also experienced decline in body mass at this time. The top-ranked body mass model showed that sex, age and capture period were important predictors of body mass at tagging (as per Supplementary Table S3 and plotted in Fig. 1).

### Survival analysis

**Figure 2:**
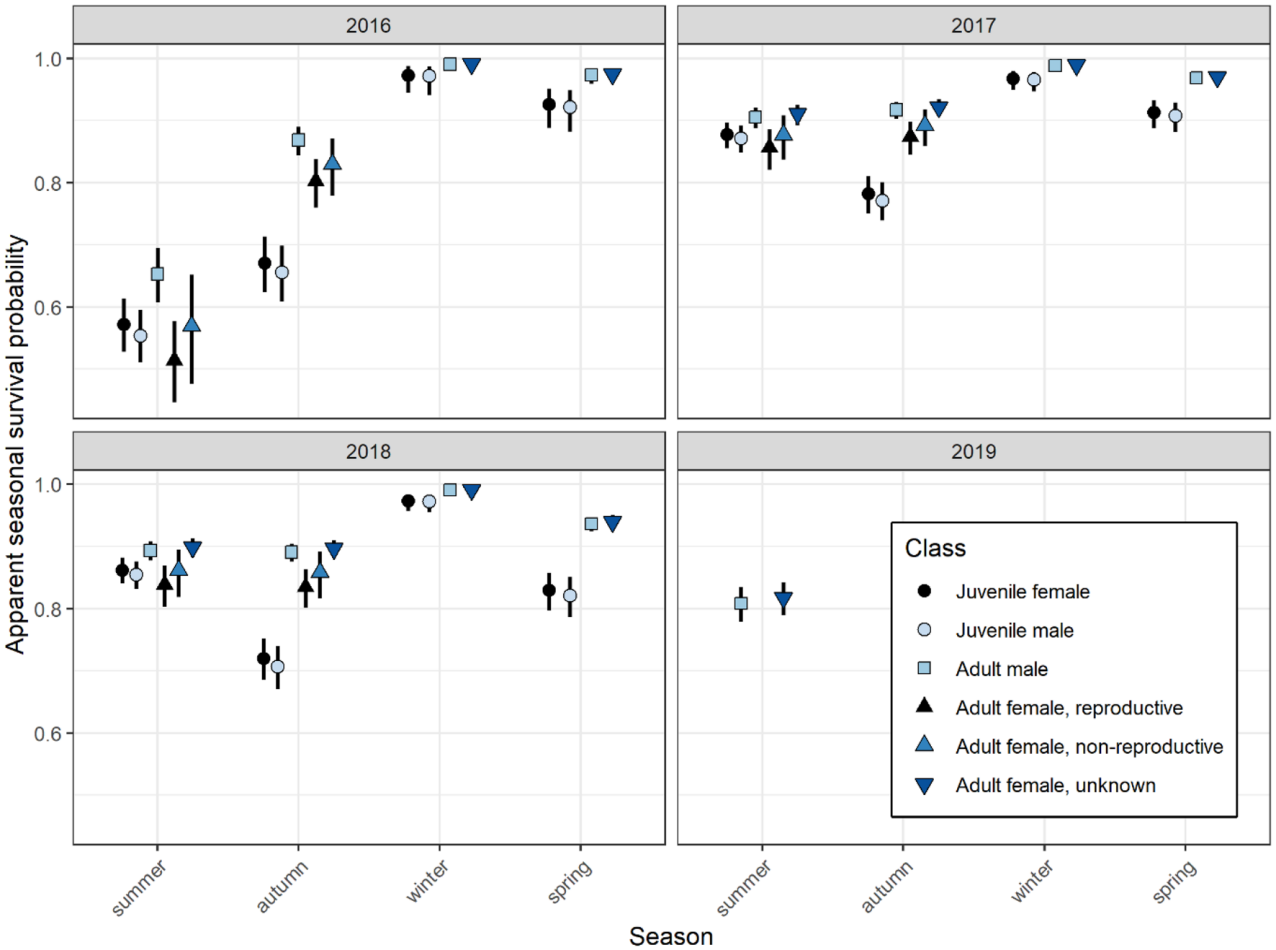
Estimates of apparent seasonal survival (i.e. the probability of surviving each three-month period), by year, for each of the defined age, sex and reproductive classes of the southern bent-winged bat at Bat Cave, Naracoorte, Australia.

The top AIC-ranked survival model included the variables age, sex, female reproductive condition and the interaction between season and year (Table 2). Survival probabilities were therefore best presented as apparent seasonal survival (i.e. the probability of surviving the three-month period) for each individual season across the study period (Fig. 2). All population cohorts had lowest apparent survival in summer and autumn, particularly in 2016 (Fig. 2 and Supplementary Table S4), which coincided with record-breaking drought conditions in the study region (a 43-month severe rainfall deficiency with rainfall totals at the lowest on record)^35^. By contrast, monthly regional rainfall for the remainder of the study period was classed as ‘very much above average’ from mid-late 2016, ‘above average’ in 2017, ranging between ‘below average’ to ‘above average’ in 2018, and ‘average’ in early 2019^35^.

**Table 2:** Model selection table for assessing best fit for apparent survival of the southern bent-winged bat at Bat Cave, Naracoorte, Australia. Shown are the deviance, AIC value, $\Delta$ AIC (difference from the ‘best’ or top-ranked model) and Akaike weight for each model. All candidate models also incorporated encounter probability ($p$) (Supplementary Fig. S1).

Model	Deviance	AIC	$\Delta$ AIC	Akaike weight
~ age + year:season + sex + reproductive	1,181,390	1,206,565	0	0.9995
~ age + year:season	1,181,413	1,206,581	16	0.0003
~ age + year:season + sex	1,181,412	1,206,583	18	0.0001
~ age + season + sex + age:season + age:sex + season:sex + age:first6months	1,181,549	1,206,718	153	< 0.0001
~ age + season + year	1,181,572	1,206,728	163	< 0.0001
~ age + season + sex + age:first6months	1,181,605	1,206,762	197	< 0.0001
~ age + season + sex + reproductive	1,181,720	1,206,877	312	< 0.0001
~ age + season	1,181,775	1,206,925	360	< 0.0001
~ age + season + sex	1,181,774	1,206,927	362	< 0.0001
~ age	1,182,694	1,207,839	1274	< 0.0001
~ age + sex	1,182,693	1,207,840	1275	< 0.0001
~ 1	1,183,065	1,208,208	1643	< 0.0001

Winter survival was high for all population cohorts (Fig. 2). Apparent survival estimated for juveniles was generally lower than for adult classes, particularly in autumn and spring—the exception being summer estimates which were even lower in reproductive females. Adult females that were assessed as reproductive (lactating) had lower survival estimates than those individuals classed as non-reproductive or for which reproductive condition was unknown (i.e. individuals tagged in previous years).

### Estimated population growth rates

The exponential rate of population growth ($r$) was calculated for each study year using a variety of different parameter assumptions (Table 3) and annual apparent survival rates from the top-ranked AIC model (Supplementary Table S5). All calculations of $r$ predicted population decline under the apparent survival rates experienced in 2016 (i.e., all *r* values were negative), with the baseline value as low as -0.603. Predicted rates of decline were markedly smaller for 2017 and 2018, though all *r* values were negative (Table 3) unless including a permanent migration rate of at least 0.1. Emigration rates in southern bent-winged bats are unknown, however, historical banding records suggest a rate less than 0.05^36^. Varying other parameters, including juvenile sex ratio, pre-volant survival and potential tag loss, made comparatively small differences to the population growth rate.

**Table 3:** Estimated exponential rates of population growth ($r$) of the southern bent-winged bat at Bat Cave, Naracoorte, Australia. Estimates of $r$ are calculated from a pre-breeding Leslie matrix parameterised using apparent annual survival rates for each study year (Supplementary Table S5, using the top-ranked model presented in Table 2) and assuming reproductive maturity of adult females at two years of age and additional parameters as specified. If parameters are not listed, they remain at the baseline parameterisation.

${\varvec{r}}$ parameters	Exponential population growth (*r*)		
	2016	2017	2018
Baseline parameterisation Sex ratio = 1:1 Permanent emigration = 0 Breeding probability = 1 Pre-volant survival = 1 Tag loss = 0	− 0.603	− 0.054	− 0.168
Juvenile sex ratio (♂:♀) = 55:45	− 0.603	− 0.074	− 0.187
Permanent emigration = 0.05	− 0.552	− 0.002	− 0.117
Permanent emigration = 0.1	− 0.498	0.052	− 0.063
Breeding probability = 0.6	− 0.603	− 0.131	− 0.238
Pre-volant survival = 0.8	− 0.603	− 0.095	− 0.207
Tag loss = 0.027	− 0.576	− 0.026	− 0.141
Tag loss = 0.05	− 0.552	− 0.002	− 0.117
Example of combined parameters #1 Permanent emigration = 0.05 Breeding probability = 0.6 Pre-volant survival = 0.8 Tag loss = 0.05	− 0.497	− 0.116	− 0.198
Example of combined parameters #2 Permanent emigration = 0.1 Breeding probability = 0.6 Pre-volant survival = 0.8 Tag loss = 0.05	− 0.441	0.002	− 0.103

## Discussion

Passive monitoring of PIT-tagged wildlife populations can provide rich data on survival rates compared with traditional recapture-based methods^4^, due to high encounter rates. Using PIT technology we found that all sex and age classes of the critically endangered southern bent-winged bat have high winter survival, with lower survival in summer and autumn. This is contrary to our expectation that survival would be lowest in winter, based on historical research on eastern bent-winged bats *Miniopterus orianae oceanensis*^32,33^. Our study adds to an increasing body of literature showing no evidence of increased mortality risk in insectivorous bats during winter^20,23,37^, except during severe or exceptional winters^14,37^, or with additional factors, such as human disturbance^38^ or white-nose syndrome^39^.

### Factors affecting survival and population decline

Apparent survival rates varied across years, with markedly lower survival in the summer and autumn of 2016. This period (January–May 2016) coincided with the last few months of a severe 43-month rainfall deficiency (with rainfall totals the lowest on record) in south-east South Australia^35^. Our general finding of lower summer survival aligns with the results of other studies using passive PIT-tag monitoring to estimate survival in temperate insectivorous bats^9,10,14^, including lower survival in drought years in big brown bats^9,10^. In contrast, a review of global survival studies in insectivorous bats has suggested higher survival rates in summer, particularly for adult females^16^; however, this finding may be confounded by sampling issues in early survival studies^8^. In temperate regions, many bats have high survival associated with winter hibernation^24,40^, whereas summer survival is more likely to be affected by factors such as adequate availability of food resources. For example, high summer rainfall is associated with increased survival estimates in adult females of the little brown myotis *Myotis lucifugus*^18^.

Our study suggests summer and autumn are key times of mortality for the southern bent-winged bat population. The probable impact of drought and human activity on prey and water availability may explain the seasonal patterns in the apparent survival estimates. The southern bent-winged bat congregates *en masse* from spring to autumn^25,41^, thereby placing concentrated demand for resources around the maternity cave. The impact of dry conditions on resource availability may be exacerbated by diminished foraging availability due to habitat loss. Forested areas and wetlands are thought to be favoured foraging habitats of the southern bent-winged bat^25^. In the geographic range of the southern bent-winged bat, over 90% of native vegetation has been cleared^25^. Most wetlands in the region (which previously regularly inundated approximately 40% of south-east South Australia) have been drained for agriculture^42^ and significant loss of wetlands has also been attributed to groundwater decline, as a result of both groundwater extraction and reduced rainfall^43^. Southern bent-winged bats are heavier than its congener the eastern bent-winged bat, despite no significant difference in forearm length. This suggests that southern bent-winged bats may have higher energy demands and may be more susceptible to adverse conditions such as drought^44^. A changing climate is bringing drier conditions to this region in south-eastern Australia, with droughts predicted to become longer and more severe^45^. As a result, warm/dry season survival rates for this critically endangered bat may worsen in the future.

Adult survival is a major driver of population dynamics in long-lived species^19^. Here, apparent survival in adults was lowest in lactating females during the summer, particularly during the drought period when reproductive females had 6% lower apparent seasonal survival compared to non-reproductive females. This is one of the first studies to show increased survival costs in lactating females, compared to non-reproductive females—though Culina et al.^46^ recently reported reduced winter survival in breeding females of Daubenton’s bat but not Natterer’s bat*.* This included a 33% lower winter survival in successful first-time breeders compared to failed first-time breeders^46^. Lactation places high energy and water demands on nursing females^47,48^. Home-ranges and foraging distance can be significantly decreased during lactation^49,50^, there is increased dependence on water sources^48,51^, and lactation typically coincides with peaks in insect activity, enabling energetic costs of nursing to be met^52^. As such, it is expected that resource limitation arising from drought disproportionately affects lactating females compared to other adult classes. Indeed, with juvenile southern bent-winged bats weighing roughly the same or more than adult females when they first start exiting the cave there must be an enormous energetic investment from adult females, presumably to increase survivability of their young. Additionally, observations of lactating females in this study suggest that juveniles continued to be nursed by their mothers for a number of weeks after they commence flying.

Apparent survival rates of juveniles were lowest in summer and autumn 2016, and juveniles generally had lower survival than adults (except for lactating females in summer) (Fig. 2). Whilst apparent survival often underestimates true survival, our summer survival rates do not include pre-volant juvenile survival (as bats were tagged after they began emerging from Bat Cave), so true summer survival of juveniles may be even lower than our results suggest. First-year individuals commonly have lower survival rates than adults^9,16,17,20^. Juvenile mortality is likely related to inexperience. In summer, juveniles newly emerging from the cave with little flying experience appear to have higher chance of injurious collisions, as observed in newly-flying juveniles at Bat Cave^53^. Inexperience may also place juveniles at increased risk of predation due to being less able to evade predators. During the study, owls were observed hunting at the entrance of Bat Cave during emergence and, on one occasion, an owl was observed roosting inside the cave (E.vH, pers. obs.). Furthermore, when young lose their mother due to mortality, as indicated by lower apparent survival in lactating females, their chance of subsequent survival is presumably low.

After weaning, juvenile mortality may be influenced by a lack of foraging experience. There was a conspicuous loss of body mass during and following the period of weaning (Fig. 1, Supplementary Table S2). Declining body mass is commonly observed in juvenile bats following the onset of flight^54^ and experience of juvenile bats appears to influence foraging success^55^. Body mass is a strong predictor of fat stores in insectivorous bats^56^ and is associated with higher survival^9,52^. For example, in big brown bats, female juveniles that did not return to monitored roosts as one-year-olds had lower body condition in late summer of their natal year than those known to survive their first year^9^. In our study, juveniles had markedly lower spring survival than adults, which could suggest that whilst winter is not a time of high mortality as initially expected, juveniles may enter spring with lower fat stores affecting subsequent spring survival. Juvenile survival estimates can be negatively biased by permanent migration from the natal site^16^. However, migration between the two major maternity populations of the southern bent-winged bat is thought to be very low, as suggested by historical banding records^36^, relative pesticide loads^57,58^ and viral diversity^27^, and natal philopatry appears to be strong in both sexes^41^.

The apparent survival estimates and derived rates of population growth in this study indicate further decline of the southern bent-winged bat population, potentially exacerbated by dry years. The highest population growth rates are estimated if permanent juvenile emigration is higher than previously assumed, although all estimates predict population decline under the apparent survival rates identified in 2016 (Table 3). There has been a severe historical decline at this maternity site since the 1960s^25^ and at least two mortality events have been observed during droughts in 1967 and late 2006^25^. Widespread clearing of natural forest and woodland vegetation did not occur in this region until after World War II. Whilst most wetland drainage in the lower southeast of South Australia occurred prior to 1970s, in the upper southeast (where Bat Cave is located) widespread drainage mostly occurred later—undertaken privately in the 1980s and then through a government program in the 1990s^42^. Therefore, habitat loss approximately coincides with reported population declines^25^. Additional pressure on foraging may include decreased prey availability due to the use of pesticides^25^. Taken together, our results suggest that challenging conditions during the breeding season are affecting population viability of the southern bent-winged bat. Given the timing of mortality, an emphasis of recovery strategies should be on boosting foraging habitat and prey availability within foraging range of maternity caves.

The southern bent-winged bat is just one of more than a third of bat species listed as threatened or data-deficient globally^15^. Many bats provide important ecosystem services such as agricultural pest control^59^. Drought, and other climatic extremes, can negatively impact bat survival^9,10,14,37^, and climate change will likely have a profound impact on population dynamics into the future^60,61^. Long-term data has linked warmer summers with increasing body mass and mortality risk in the Bechstein’s bat *Myotis bechsteinii*^62^*.* An increasingly drier and hotter climate has also been linked to increasing male bias in juvenile sex ratios of several bat species^63^. In our study, the male bias of juveniles in 2017 differed significantly from the expected 1:1 sex ratio. Male-biased sex-allocation as a result of drought is a plausible explanation because conception occurs immediately after mating in late autumn^64^ and drought-breaking rainfall did not occur until the winter of 2016^35^. Further sampling is required to test the relationship between climate and sex ratios in southern bent-winged bats, an aspect of clear importance for predicting long-term population dynamics.

### Use of PIT technology and CMR analyses for survival-rate estimation

Low physical recapture rates in bats provides challenges for the use of traditional mark-recapture approaches. Here, the use of passive monitoring at a roost provided an abundance of re-sight data, resulting in very high encounter probabilities with reduced disturbance^5^. For example, in this study even during periods of relatively lower daily encounter probabilities, such as over winter (Supplementary Fig. S1), $p$ for a tagged individual was close to 1 when calculated across a 3-month season. However, challenges also occur in using PIT-data for survival analyses. Continuous data violates the assumption of instantaneous sampling in CJS models and can bias resulting survival estimates, particularly if sampling periods are long^65^. The Barker joint model outperforms CJS models in estimating survival from continuous data when using monthly and ten-day sample bins^66^. Increasing sample size in these comparisons did not overcome bias in CJS models, because increasing the number of individuals resulted in overly narrow confidence intervals. A drawback is that the Barker joint model is prohibitively complex for use in many real-life applications^66^. Additionally, the Barker model requires primary capture periods dispersed between the continuous mark-resight data, and this may not always be possible or appropriate for reasons such as disturbance to vulnerable populations, or very low recapture rates for elusive species that are hard to capture, such as bats. During trapping we recaptured just 52 of our 2966 tagged individuals^67^.

Our approach to the problem of instantaneous sampling was to choose daily time intervals for binning detection data, to ensure the data was as close to instantaneous as possible (whilst still ecologically and practically reasonable). However, this study was based on a dataset of in excess of 1.6 million unique individual detections. Fitting models for this quantity of data is time consuming, requires the use of high-performance computing (HPC) clusters and it becomes increasingly difficult to converge more complex models (for example with higher numbers of interacting variables). An alternative approach by Reusch et al.^14^ used simpler mixed-effects logistic regression (with an individual-specific random intercept) to examine bat survival from continuous PIT-tag data, but limited explanation was provided on how this approach compares to traditional CMR models. There is a need to develop robust methods for the analysis of increasingly large, continuous mark-resight datasets emerging from the use of new technologies. These solutions should balance the challenges that can arise from larger datasets, such as analysis time, computing power and false confidence, with usability in applied ecology for informing species management.

Another assumption of CJS models is that tags are not lost: therefore, tag loss can negatively bias estimates^68^. PIT-tag loss predominantly occurs soon after tagging due to tags working their way out of the insertion hole^69^ and can be minimised by using surgical adhesive on the injection site^70^, as in this study. Double-tagging (with a second alternative marking method) can allow for estimating tag loss rates for interpreting subsequent survival results, but requires continued physical recapture to confirm tag loss^69^. We decided that ongoing trapping to confirm tag loss rates posed too much disturbance for this critically endangered bat. Although the ‘first6months’ variable (which fitted separate survival estimates for the first six months after an individual’s tagging and for its remaining resight history) did not provide the best fit for the data (Table 2), survival results from after six months of tagging showed similar seasonal results of lower summer and autumn survival. Thus, the seasonal survival pattern identified could not be explained solely by tag loss or other marking effects (Supplementary Fig. S2).

PIT-tagging appears to be a safe marking method for bats, with no evidence of effects on body condition, reproductive success, infection or other detrimental effects^67,71,72^. It is unlikely that apparent survival differences are due to PIT-tagging affecting survivability, but we cannot preclude this possibility. Ongoing research and transparent documentation of tag loss rates and marking effects in a range of species is critical to inform data interpretation and to ensure the marking procedures continue to be informed by the best available knowledge to minimise disturbance to wildlife.

Our CMR approach using large-scale passive detection has provided rich data on apparent survival rates of the southern bent-winged bat, including sex, age and reproductive classes across all seasons in consecutive years, as well as population growth estimates. This information will be critical for developing targeted recovery actions to reduce extinction risk in this critically endangered species, and has particularly highlighted the need for increased foraging resources around maternity caves. In addition, we showcase the potential of this approach for providing complex, robust analysis on population dynamics for wildlife species to address knowledge gaps into the future, such as the demographic responses to climate change^73^, and the large proportion of species with unknown population trends worldwide, particularly for bats^74^.

## Methods

### PIT-tagging and data collection

Southern bent-winged bats were trapped and PIT-tagged at Bat Cave within the Naracoorte Caves National Park, South Australia, which serves as a maternity and congregation site from spring to autumn^41^. Trapping occurred over six nights in 2016, and four nights in each of 2017 and 2018, at the end of the breeding season (January and February) by which time juveniles born in November were emerging from the cave to forage at night. Bats were trapped with Austbat harp traps (Faunatech, Mount Taylor) surrounding the cave entrance. Trapping continued throughout the night, catching bats as they left or re-entered the cave. Both females and males congregate at Bat Cave, so both sexes could be tagged. There was no targeting of one sex over the other, and hence the tagged samples reflect the sex ratio of trapped individuals. A potential male bias (from the expected 1:1) in juvenile sex ratios in 2017 was tested for statistical significance using a Chi-squared test.

Individual covariates were recorded for each PIT-tagged bat, including sex, body mass and age. Age was described as juvenile (first year) or adult based on the respective presence or absence of a cartilaginous core at the metacarpal-phalangeal joints^75^. The reproductive condition of adult females was classified as pre-parous, lactating or post-lactating through examination of the nipples^76^. PIT-tags were subcutaneously injected dorsally using a sterilised 12-gauge needle and applicator (Biomark MK10 implanter and N125 needles in 2016, Biomark MK 25 Implant Guns and HPT12 Pre-load Trays in 2017–18). The injection site was sealed with a drop of surgical adhesive (3 M™ VetBond™) to minimise tag loss, and allowed to dry prior to release^67^. All PIT-tags (Biomark HPT 12) were checked for correct function using a hand-held PIT-tag scanner (Trovan LID560 and Biomark 601) both before and after insertion. During handling and tagging, bats typically remained calm and were able to fly within minutes of the procedure. Recaptured individuals were in good physical condition, with no sign of infection or other detrimental effects^67^. Linear models were used to explore the relationship between capture periods, demographic variables, and body mass recorded at the time of tagging (Supplementary Table S3).

Tagged bats were monitored by using a large PIT-tracking system (Biomark IS1001) which employed a 2 × 5 m loop antenna as described in van Harten et al.^5^*,* installed within Bat Cave, which detected tagged individuals in real time as they flew through the cave passage. When the system was working optimally, there was a large read-range before and after the antenna plane and high detection success^5^. Data files were recorded directly to USB flash drives plugged into the data logger board of the Biomark IS1001. Data were collected from the cave regularly (approximately monthly) over a 37-month period by manually retrieving the flash drives.

### Survival analyses

To prepare the data for analysis, we derived capture-resight histories for each of the 2966 PIT-tagged bats to produce a binary response variable (undetected/detected) for each individual across each day of the 1120-day study period, with a ‘day’ being defined as the 24 h between successive middays. Accumulative age functions were incorporated to allow juvenile bats to age appropriately (i.e., bats tagged as juveniles were classified as adults at 1 year of age). As reproductive status in adult females could be determined only by physical examination during PIT-tagging, these individuals were defined as ‘reproductive’ (i.e. lactating) or ‘non-reproductive’ for the summer and autumn following tagging, and then pooled into an ‘unknown’ reproductive category for all subsequent times. We then modelled daily survival using Cormack-Jolly-Seber (CJS) models which were fitted using the R package ‘RMark’^77^. CJS models allow estimation of apparent survival probabilities ($\varphi$) whilst also accounting for (potentially variable) encounter probabilities ($p$).

Given the impact of environmental noise on antenna performance^5^, and the seasonal behaviour and movement patterns of the southern bent-winged bat^41^, we modelled encounter probability with flexible spline functions such that

$$p \sim s\left(yday, k=5\right)+s(noise, k=2)$$

where $yday$ is the day of year, $noise$ is the average environmental noise (summation of unwanted signal being received by the PIT tracking system, %) over the course of that day, and $k$ is the dimension of the spline. The basis functions for the splines were calculated with the R package ‘mgcv’, and a cubic regression spline was assumed for the day-of-year effect to ensure continuity in the response between the first and last day of year.

We then developed a candidate set of models for daily survival probability $(\varphi )$ that included different covariates including age, sex, season, year and reproductive condition (for adult females only), as well as possible interaction terms. When modelling the effect of both season and year, we coded December as a component of summer in the following year (e.g. December 2016 data were coded as summer 2017). We also tested a variable $first6months$ which fitted separate survival estimates for the first six months after an individual’s tagging and for the remainder of the re-sight history, to explore any possible marking effects. Model selection was undertaken by comparing Akaike information criterion (AIC) for each alternative model. The AIC includes a penalty for increasing complexity (i.e. number of parameters) in the model^78^. The ‘best’ or top-ranked model is the one with the lowest AIC value.

### Estimation of population growth rates

In this study, we use ‘population’ to refer to the colony of southern bent-winged bats that occupy the Bat Cave maternity site. To estimate the exponential rate of population growth (*r*), we constructed pre-breeding Leslie matrices^79^. To achieve this, we initially used annual survival-rate estimates for each age, sex and reproductive class (derived from daily survival estimates from the top AIC-ranked model), assumed a female age of reproductive maturity of two years (as described for eastern bent-winged bats^80^ and observed in this study as reported above) and fertility estimates of one offspring per adult female per year^81^ as baseline parameterisation. We then calculated the effect on population growth rates under different demographic assumptions, including differing juvenile sex ratios (from observations in this study), potential emigration rates, breeding probabilities and tag loss. Two potential rates of tag loss were tested: 2.7% which was calculated for the Gould’s wattled bat *Chalinolobus gouldii* in a double-tagging experiment using the same PIT-tagging procedures as this study^69^; and 5%, the approximate proportion of bats not detected more than 10 days after tagging during this study in 2017 and 2018^5^ which provides an estimate of the maximum tag loss over this period.

### Ethics approval

Animal capture, handling and data collection were approved by the La Trobe University Animal Ethics Committee (Project Number AEC15-67) and were carried out in accordance with relevant guidelines and regulations prescribed by the South Australian Department of Environment and Water (Research Permit Number U26453). This study was carried out in compliance with the ARRIVE guidelines on animal research.

## Supplementary Information

Supplementary Information.

## Data Availability

The datasets generated during and/or analysed during the current study are available from the corresponding author on reasonable request.
