# Supplementary material for: Novel passive detection approach reveals low breeding season survival and apparent lactation cost in a critically endangered cave bat
Source: Sci Rep. 2022 May 5;12:7390. doi: 10.1038/s41598-022-11404-4 (PMC9072322; doi:10.1038/s41598-022-11404-4)
Supplement: Supplementary file 1 — Supplementary Information. [file 41598_2022_11404_MOESM1_ESM.pdf]

Novel passive detection approach reveals low breeding season survival and apparent lactation cost in a critically endangered cave bat

Scientific Reports

Emmi van Harten[1*], Ruth Lawrence[2], Lindy F. Lumsden[3], Terry Reardon[4], Thomas A. A. Prowse[5]

[1]Department of Ecology, Environment and Evolution, Research Centre for Future Landscapes, La Trobe University, Bundoora, Victoria 3086, Australia, e.vharten@latrobe.edu.au *Corresponding author.

[2]Department of Geography, University of Melbourne, Parkville, Victoria 3010, Australia

[3]Arthur Rylah Institute for Environmental Research, Department of Environment, Land, Water and Planning, Heidelberg, Victoria 3084, Australia

[4]South Australian Museum, Adelaide, South Australia 5001, Australia

[5]School of Biological Sciences, The University of Adelaide, Adelaide, South Australia 5005, Australia

Supplementary information

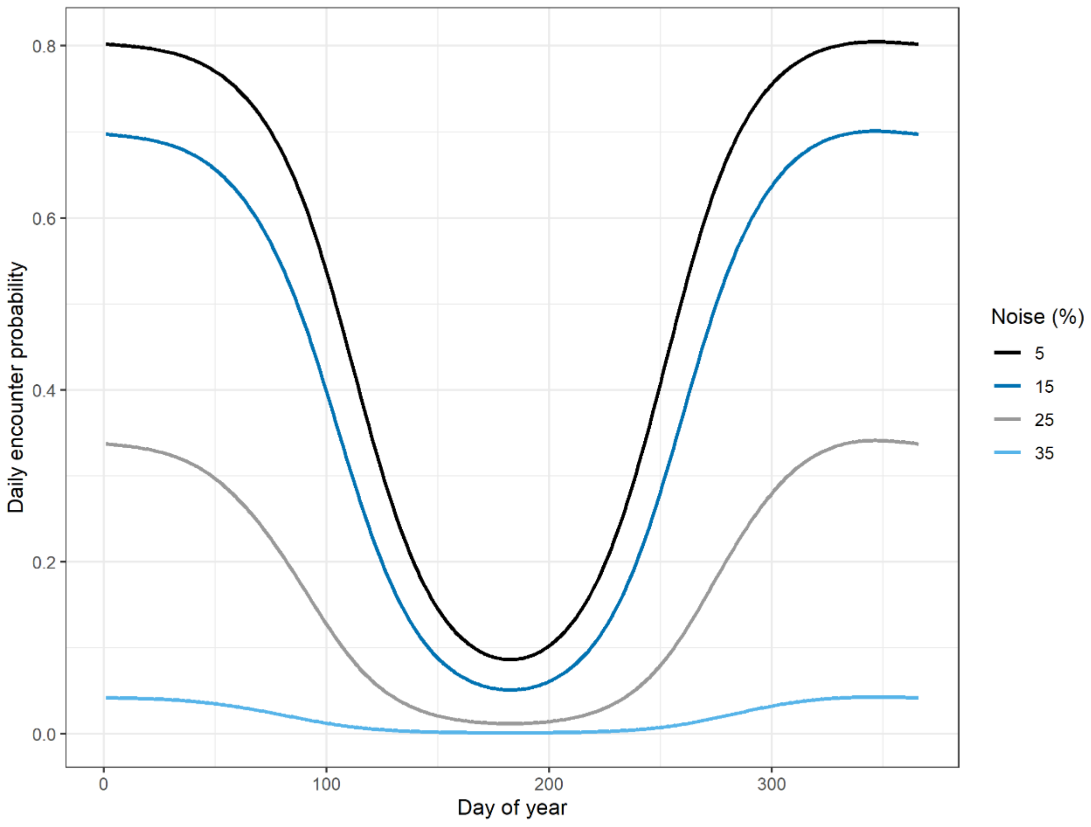

Supplementary Figure S1. Daily encounter probability of southern bent-winged bats, showing the modelled effects of day of year ($yday$) when $noise$ = 5%, 15%, 25% and 35%. Encounter probability was included in the survival models in this study to account for variation in seasonal behaviour of the bats and environmental 'noise' perceived by the antenna system. Daily average noise levels typically ranged between 5–15%.

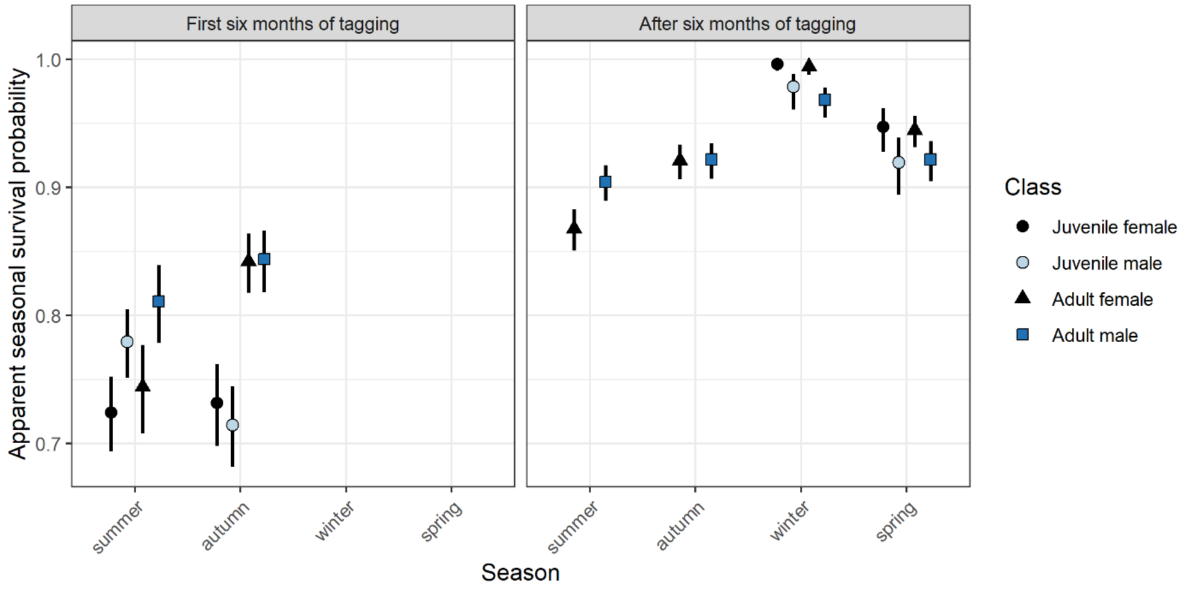

Supplementary Figure S2. Apparent seasonal survival estimates for the first six months of PIT-tagging and subsequent survival of southern bent-winged bats. Although models using the ‘first-six-months’ variable did not provide best fit compared to the final model, the results from after six months of tagging (when any tag loss is presumed to have negligible effect on survival estimates), show that subsequent survival is still lowest in summer and autumn and therefore this seasonal pattern could not be explained solely by tag loss or potential marking effects. It is important to note that estimates for the first six months of tagging include the summer and autumn of 2016, which was evidently a challenging season with higher mortality rates coinciding with drought. Therefore, the apparent survival rates in the first six months should not be taken as an indicator of marking effects on the population.

Supplementary Table S1. Lactation status for 662 captures (including recaptures) of adult female southern bent-winged bats. Shown are the number assessed as lactating, not lactating (i.e. either pre-parous or post-lactating), and the proportion of adult females lactating (given as a % of the total assessed for that capture period). January lactation rates over the study suggest that the majority of adult females breed each year. A capture period on 3 February 2017 showed just 28.5% of adult females were lactating, suggesting that juveniles were being weaned at this time.

Capture period		Lactating	Not lactating
year	date		
2016	8 Jan	132 (82%)	29
2016	15 Jan	47 (100%)	-
2016	19 Feb	-	2
2017	13 Jan	50 (73.5%)	18
2017	3 Feb	35 (28.5%)	88
2018	12 Jan	110 (62.5%)	66
2018	19 Jan	72 (84.7%)	13

Supplementary Table S2. Body mass (g) of southern bent-winged bats (n) at each capture period (with date of the first capture night) at Bat Cave, Naracoorte, Australia.

**Capture period**		**Juvenile females**				**Juvenile males**				**Adult females**				**Adult males**			
**year**	**date**	**n**	**mean**	**s.e.**	**range**	**n**	**mean**	**s.e.**	**range**	**n**	**mean**	**s.e.**	**range**	**n**	**mean**	**s.e.**	**range**
2016	8 Jan	10	16.2	0.19	15.4-17.3	5	17.0	0.31	15.8-17.5	188	16.2	0.06	13.3-19.0	156	16.7	0.07	12.6-18.9
2016	15 Jan	189	16.1	0.06	14.2-18.4	198	16.7	0.06	14.0-18.8	55	16.1	0.11	14.5-18.9	20	16.3	0.19	14.4-17.6
2016	19 Feb	83	13.1	0.11	10.8-15.8	68	13.4	0.11	11.0-15.7	-	-	-	-	-	-	-	-
2017	13 Jan	254	16.1	0.05	13.6-18.2	311	16.7	0.04	14.7-18.6	64	16.2	0.08	14.6-17.5	20	17.1	0.16	15.9-19.0
2017	3 Feb	38	15.8	0.12	14.5-18.1	47	16.3	0.13	14.4-19.0	124	17.3	0.18	13.8-22.5	141	17.5	0.11	14.4-24.8
2018	12 Jan	44	16.5	0.14	14.3-18.2	38	17.1	0.15	14.5-19.0	174	16.7	0.06	12.7-18.7	148	17.3	0.07	15.2-20.0
2018	19 Jan	225	16.1	0.05	14.1-18.5	211	16.6	0.06	14.1-19.2	81	15.9	0.08	14.0-17.9	74	16.7	0.10	13.5-19.4

Supplementary Table S3. Model selection table for assessing best fit for body mass of the southern bent-winged bat at Bat Cave, Naracoorte, Australia. Shown are the deviance, AIC value, ΔAIC (difference from the 'best' or top-ranked model) and Akaike weight for each model. Only juveniles were tagged in one capture period – this was accounted for by including age as an interacting term with each time variable. Lactation period was defined by lactation rates in adult females as 'peak-lactation', 'weaning' and 'post-lactation'.

**Model**	**Deviance**	**AIC**	**ΔAIC**	**Akaike weight**
body mass ~ sex + age + age : capture period	2563	8014	0	>0.9999
body mass ~ sex + age + age : lactation period	2652	8100	86	<0.0001
body mass ~ sex + age + age : day of year	2850	8311	297	<0.0001
body mass ~ sex + age	4132	9408	1394	<0.0001
body mass ~ age	4399	9592	1578	<0.0001
body mass ~ sex	4458	9631	1617	<0.0001

Supplementary Table S4. Apparent daily and seasonal survival probabilities (phi), with upper and lower confidence levels (95%), for southern bent-winged bats at Bat Cave, Naracoorte, Australia, using the top AIC-ranked survival model.

**Year**	**Season**	**Class**	**Daily survival probability**			**Seasonal survival probability**		
			**phi**	**lcl**	**ucl**	**phi**	**lcl**	**ucl**
2016	Summer	Adult female, non-reproductive	0.993846697	0.991909	0.995323	0.569151133	0.476229	0.651753
		Adult female, reproductive	0.992745604	0.991225	0.994004	0.514360219	0.447195	0.577435
		Adult male	0.995347331	0.99456	0.996021	0.653222014	0.60769	0.69486
		Juvenile female	0.986885884	0.985017	0.988524	0.572031273	0.527951	0.61362
		Juvenile male	0.986157463	0.984253	0.987835	0.554435932	0.51089	0.595766
	Autumn	Adult female, non-reproductive	0.997961485	0.997264	0.998481	0.829998103	0.778694	0.870411
		Adult female, reproductive	0.997594924	0.997004	0.99807	0.80261673	0.760327	0.838256
		Adult male	0.998460182	0.998144	0.998723	0.868738602	0.843981	0.889829
		Juvenile female	0.995635008	0.994847	0.996303	0.670688315	0.623933	0.713025
		Juvenile male	0.995390286	0.994576	0.996083	0.655801117	0.608563	0.698814
	Winter	Adult female, unknown	0.999901367	0.999789	0.999954	0.991033589	0.980934	0.995795
		Adult male	0.999895812	0.999777	0.999951	0.990530985	0.979885	0.995555
		Juvenile female	0.999703873	0.99937	0.999861	0.973318346	0.944047	0.987379
		Juvenile male	0.999687199	0.999334	0.999853	0.971837106	0.940955	0.986682
	Spring	Adult female, unknown	0.999718759	0.999565	0.999818	0.974642628	0.961053	0.983532
		Adult male	0.999702923	0.999541	0.999808	0.973233879	0.958955	0.982591
		Juvenile female	0.999155935	0.998703	0.999451	0.925791534	0.888228	0.951081
		Juvenile male	0.999108436	0.998628	0.999421	0.921781356	0.882203	0.948452
2017	Summer	Adult female, reproductive	0.998305611	0.997839	0.998672	0.856543526	0.820732	0.885707
		Adult female, non-reproductive	0.998564011	0.998049	0.998943	0.877026561	0.836664	0.90797
		Adult female, unknown	0.998973237	0.998754	0.999154	0.910460744	0.892361	0.925646
		Adult male	0.998915467	0.998692	0.999101	0.905665616	0.887347	0.92114
		Juvenile female	0.996923052	0.996316	0.99743	0.877749478	0.855434	0.896835
		Juvenile male	0.996750308	0.996115	0.997282	0.871336974	0.848138	0.891227

	Autumn	Adult female, non-reproductive	0.99874734	0.99833	0.99906	0.891851872	0.858487	0.917732
		Adult female, reproductive	0.99852188	0.99815	0.998819	0.873654186	0.844475	0.897696
		Adult female, unknown	0.999104368	0.99893	0.99925	0.921438669	0.906857	0.933823
		Adult male	0.99905397	0.99888	0.999201	0.917204067	0.902705	0.929628
		Juvenile female	0.997315315	0.996862	0.997703	0.782332897	0.750553	0.810591
		Juvenile male	0.997164531	0.996698	0.997565	0.771605737	0.739326	0.800446
	Winter	Adult female, unknown	0.999881047	0.999811	0.999925	0.989196238	0.982873	0.993193
		Adult male	0.999874348	0.999801	0.999921	0.988591244	0.981955	0.992796
		Juvenile female	0.99964288	0.999436	0.999774	0.967910806	0.949751	0.979579
		Juvenile male	0.999622773	0.999404	0.999761	0.966134681	0.946993	0.978443
	Spring	Adult female, unknown	0.999667138	0.999561	0.999748	0.970057926	0.9607	0.977214
		Adult male	0.999648397	0.999538	0.999733	0.968398671	0.958659	0.975873
		Juvenile female	0.999001113	0.998694	0.999236	0.912783601	0.887513	0.932596
		Juvenile male	0.998944911	0.99862	0.999194	0.90810642	0.88151	0.928977
2018	Summer	Adult female, non-reproductive	0.998367614	0.997812	0.998782	0.861414899	0.818725	0.894706
		Adult female, reproductive	0.998073942	0.997597	0.998456	0.838582105	0.802803	0.868412
		Adult female, unknown	0.998832743	0.998637	0.999	0.89884258	0.882919	0.91271
		Adult male	0.998767079	0.998567	0.998939	0.893462855	0.877302	0.90761
		Juvenile female	0.996503012	0.995896	0.997021	0.862236557	0.84028	0.881396
		Juvenile male	0.996306774	0.995658	0.996859	0.855081134	0.831842	0.875358
	Autumn	Adult female, non-reproductive	0.998324539	0.997779	0.998736	0.858027752	0.81628	0.890931
		Adult female, reproductive	0.998023132	0.997575	0.998389	0.834692922	0.801136	0.863082
		Adult female, unknown	0.998801927	0.998615	0.998964	0.896313893	0.881095	0.909687
		Adult male	0.998734532	0.998547	0.998898	0.890808115	0.875694	0.904187
		Juvenile female	0.996410911	0.995871	0.99688	0.720133814	0.685382	0.751772
		Juvenile male	0.996209524	0.995641	0.996704	0.706964016	0.671052	0.739749
	Winter	Adult female, unknown	0.999903559	0.999842	0.999941	0.991232003	0.985644	0.994651
		Adult male	0.999898128	0.999833	0.999938	0.990740469	0.984853	0.994346
		Juvenile female	0.999710454	0.999524	0.999824	0.973903519	0.957441	0.984052
		Juvenile male	0.99969415	0.999496	0.999814	0.972454298	0.955044	0.983182

	Spring	Adult female, unknown	0.999319156	0.999177	0.999437	0.93970366	0.927561	0.949867
		Adult male	0.999280836	0.999132	0.999404	0.936418949	0.923768	0.947032
		Juvenile female	0.997958274	0.997519	0.99832	0.829754243	0.797028	0.857686
		Juvenile male	0.997843523	0.99737	0.998232	0.821087194	0.78627	0.850783
2019	Summer	Adult female, unknown	0.997793435	0.997416	0.998116	0.817332243	0.789592	0.841791
		Adult male	0.997669441	0.997269	0.998011	0.808109632	0.779019	0.833791

Supplementary Table S5. Apparent annual survival probabilities used for calculating rates of r (exponential population growth) of southern bent-winged bats at Bat Cave, Naracoorte, Australia, for each study year, calculated from the top AIC-ranked model.

**Class**	**Annual survival probability**		
	**2016**	**2017**	**2018**
Adult female, non-reproductive	0.456	0.751	0.688
Adult female, reproductive	0.399	0.718	0.652
Adult male	0.547	0.795	0.738
Juvenile female	0.346	0.607	0.502
Juvenile male	0.326	0.590	0.483
